# Bisphosphonates and atrial fibrillation: Bayesian meta-analyses of randomized controlled trials and observational studies

**DOI:** 10.1186/1471-2474-10-113

**Published:** 2009-09-21

**Authors:** Anselm Mak, Mike WL Cheung, Roger Chun-Man Ho, Alicia Ai-Cia Cheak, Chak Sing Lau

**Affiliations:** 1 Division of Rheumatology, Department of Medicine, Yong Loo Lin School of Medicine, National University of Singapore, Singapore; 2 Department of Psychology, Faculty of Arts and Social Sciences, National University of Singapore, Singapore; 3 Department of Psychological Medicine, Yong Loo Lin School of Medicine, National University of Singapore, Singapore; 4 Division of Rheumatology, Department of Medicine and Therapeutics, Ninewells Hospital & Medical School, University of Dundee, Dundee, UK

## Abstract

**Background:**

Occurrence of atrial fibrillation (AF) amongst bisphosphonate users has been increasingly reported but results are conflicting. We performed a Bayesian meta-analysis to address the possible association between the occurrence of AF and bisphosphonate use and estimated the posterior probability of development of AF with bisphosphonate use.

**Methods:**

Randomized controlled trials (RCTs) evaluating the efficacy and safety of bisphosphonates for treating and preventing osteoporosis, and observational studies investigating the incidence of AF amongst bisphosphonate users, were searched in electronic databases. We pooled the effect size with Bayesian meta-analysis for odds ratio (OR) and calculated its posterior probability of development of AF in bisphosphonate users for RCTs and observational studies, reported with the 95% credible interval (CI).

**Results:**

Of 1751 potentially relevant citations initially retrieved, 4 RCTs and 2 reports of RCTs, and 3 observational studies were included for this meta-analysis. On pooling the RCTs, there was a non-significantly higher risk of overall (OR 1.184, 95% CI 0.837-1.656) and serious AF (OR 1.590, 95% CI 0.613-3.751) in bisphosphonate-treated patients. Combining data of observational studies also revealed a non-significantly higher risk of AF in bisphosphonate users (OR 1.251, 95% CI 0.980-1.732). Using Bayesian meta-analysis based on the effect size of observational studies as the prior, the posterior probability of OR>1.2 in the development of AF amongst bisphosphonate users in the RCTs was 0.484. Egger's regression demonstrated no notable publication bias in all the analyses.

**Conclusion:**

The current meta-analysis revealed no evidence of a higher risk of AF associated with bisphosphonate use. Nevertheless, based on Bayesian meta-analysis with the effect size of the observational studies as the prior, the posterior probabilities of development of AF was found to be 0.484 if the risk of AF was estimated to be more than 20%. The results of the current meta-analysis thus offer clinicians the practical probability of development of AF in patients who take bisphosphonates for the treatment of bone loss and corticosteroid induced osteoporosis.

## Background

Bisphosphonates are currently the first-line therapeutic agents for treating postmenopausal women and men with osteoporosis, and patients with corticosteroid-induced osteoporosis [1-5]. While the vast majority of randomized controlled trial (RCTs) and observational studies demonstrated that bisphosphonates are both efficacious and promisingly safe in preserving bone density, preventing fragility fractures and reducing mortality [6-11], a few unexpected adverse effects, such as osteonecrosis of the jaw, hepatotoxity, auditory hallucination and visual disturbance have been increasingly described in the literature [12-15]. More recently, a RCT evaluating the efficacy and safety of zoledronic acid in postmenopausal women with osteoporosis demonstrated an unexpectedly higher risk of atrial fibrillation (AF) in patients who received the yearly intravenous zoledronate infusion compared to the control group [16]. In view of this concern, data from the Fracture Intervention Trials (FIT) [17] and a few RCTs involving risedronate [18] have recently been reviewed and the incidence and risk of AF regarding bisphosphonate use were analyzed. Review of these data illustrated a non-significant increase in the risk of AF in those who received bisphosphonates than those who received placebo [17,18]. Besides RCTs, three population based case-control observational studies addressing the potential hazard of AF in bisphosphonate use have recently been published [19-21]. Two of them demonstrated a significantly higher risk of AF even after adjustment for potential confounders [19,21] while the other revealed a non-significant trend for development of AF amongst bisphosphonate users [20]. Given the relatively insufficient sample sizes in these studies to observe such a rare event which was not anticipated at the outset, inconsistency of results between these studies is not surprising. To overcome this issue while we are awaiting data of larger RCTs with longer observational period, it is possible to generate an estimate of the effect size by pooling the data of the currently available studies by validated statistical methods. This strategy will increase the sample size and hence the power to detect a difference between bisphosphonate users and non-users with regard to the risk of development of AF.

Meta-analysis is a statistical procedure for combining results of several studies to generate a single estimate of the major effect with enhanced precision and it is regarded as a powerful tool for summarizing inconsistent findings from different studies [22]. In the present meta-analysis we tried to address whether an excess risk of AF exists amongst bisphosphonate users by pooling data from all currently available RCTs and large observational studies. In addition, we calculated the posterior probability of development of AF occurred in a number of clinically practical risks amongst bisphosphonate users based on the risk observed in observational studies, by using Bayesian meta-analysis.

## Methods

We performed a literature search by using the combinations of the relevant keywords "bisphosphonates", "zoledronate", "ibandronate", "alendronate", "risedronate", "etidronate", "pamidronate", "clodronate", "adverse events", "atrial", "atrial flutters" and "atrial fibrillation" to identify RCTs and observational studies in full publications in the English language from different computerized databases: MEDLINE (up to April 2009), EmBASE (up to April 2009) and the Cochrane Centre Register of Controlled Trials (up to April 2009). Abstracts published in major international rheumatology conferences (Annual European Congress of Rheumatology, ACR/ARHP Annual Scientific Meeting and Congress of the Asia Pacific League of Associations for Rheumatology) over the past 10 years were manually searched. We also scanned the articles from the bibliographies of retrieved trials and review articles. We excluded trials which evaluated the efficacy and safety of bisphosphonates other than for bone loss, osteoporosis, fractures and corticosteroid-induced osteoporosis. We pre-determined that the corresponding authors should only be contacted by the first author (A.M.) for essential information which were lacking in the published articles.

For RCTs, all trials which randomly assigned patients to receive either a bisphosphonate (zoledronate, ibandronate, alendronate, risedronate, etidronate, pamidronate or clodronate) or placebo for treatment and/or prevention of bone loss and osteoporosis were retrieved for evaluation. We included those RCTs for analyses if they met the following criteria: (i) compare a bisphosphonate with a placebo (ii) administer concomitant therapy with adequate calcium and vitamin D in both groups and (iii) report the occurrence of AF.

For observational studies, only those which involved control groups without exposure to bisphosphonates and reported the proportion of patients with AF on both bisphosphonate exposed and unexposed groups were eligible because our aim is to identify the risk of AF from bisphosphonates exposure.

Two investigators (A.M. and A.A.C) independently assessed the papers generated for relevancy. Papers with the following exclusion criteria were excluded: (i) not having English abstract; (ii) not reporting the occurrence of AF and (iii) not evaluating the efficacy and safety for the prevention and/or treatment of bone loss and fragility fractures. Data were independently extracted into a standard electronic data extraction form. Any discrepancies were resolved by consensus. If consensus could not be reached, the principal investigator (A.M.) would make the final decision for trial eligibility and data extraction. The second author (M.W.C) provided expert biostatistical advice while the senior author (C.S.L) was the advisor of this meta-analysis.

The quality of the RCTs was assessed based on a standard scoring system suggested by Jadad [23]. The assessment is based on (i) whether the randomization method is appropriate, (ii) whether double blindness is mentioned in the trial and whether it is appropriate and (iii) whether the number of patients of and the reasons for withdrawal and dropouts are clearly stated. The score ranges from 0 to 5 with higher scores denote better quality of a trial.

We performed Bayesian random-effects meta-analysis with non-informative prior (mu~dnorm(0, 0.0001) and precision~dgamma(0.001, 0.001)) to pool the effect sizes of the RCTs and observational studies separately and summarized as odds ratio (OR) of development of AF between bisphosphonate users and non-bisphosphonate users/placebo groups, with their corresponding 95% credible intervals (CI) [24]. The use of non-informative prior implies that we do not have strong belief on the values of the pooled effect size in the Bayesian meta-analysis. Results based on Bayesian meta-analysis with non-informative prior and classical meta-analysis are very similar. One hundred thousand iterations were used in the Bayesian analysis. There are two advantages of using Bayesian meta-analysis in the current study. First, as suggested by Sutton et al [24] and Ades et al [25], it is possible to combine findings of RCTs and observational studies by using the estimates from the observational studies as the prior. This provides an overall estimate on all RCTs and observational studies. This estimate is more precise than that from the RCTs and observational studies. Second, Bayesian meta-analysis allows us to estimate the posterior probability of the effect size that is larger than a particular value. This helps us to estimate the probability of the practical clinical significance that is larger than a particular OR. Since the results of Bayesian meta-analysis are sensitive to the priors, we used several priors to check the sensitivity of the findings.

For each meta-analysis we assessed the heterogeneity with the use of the I^2 ^statistics which describes the percentage of total variation across studies caused by heterogeneity rather than chance. High values of I^2 ^suggest increased heterogeneity. Publication bias was examined statistically by Egger's regression test.

All statistical analyses in this meta-analysis were performed using the WinBUGS 1.4.3 [26] and R [27].

To ensure the quality of the meta-analyses, both the MOOSE (meta-analysis of observational studies in epidemiology) and QUOROM (quality of reporting of meta-analysis) guidelines were adhered where applicable [28,29].

## Results

### Search results

We initially searched for RCTs on respective bisphosphonate and retrieved 1,680 citations through the electronic databases as described in the Method section. After excluding review articles (n = 43), author replies (n = 26), case reports (n = 18), non-English articles (n = 3), non-RCTs (n = 503), unrelated clinical trials (n = 571), RCTs not reporting AF (n = 505) and experimental studies (n = 8), 2 RCTs and 1 short communications which included another 2 RCTs were eligible for the meta-analysis. Amongst these 4 RCTs, two were testing the efficacy and safety of zoledronate (the HORIZON Pivotal Fracture Trial and the HORIZON Recurrent Fracture Trial) [11,16] and the other two were published as short communication [17] which consisted of data of the two Fracture intervention Trials [30,31]. We also identified a communication published as a short report comprising data of 5 RCTs testing the efficacy and safety of risedronate [18]. We further identified one abstract comprising 4 RCTs which reported the incidence of AF in ibandronate users presented in the Annual European Congress of Rheumatology in June 2008 [32].

**Figure 1:**
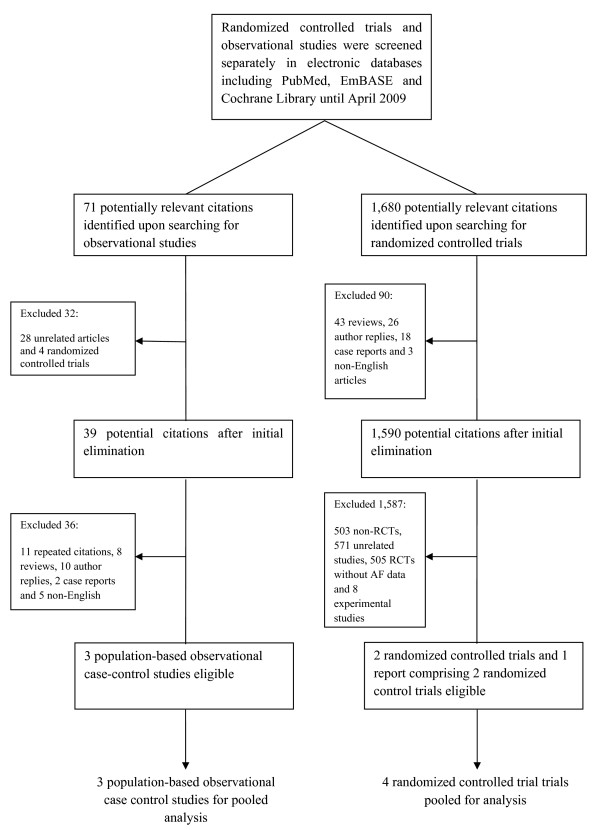
**Summary of the literature search**.

We subsequently performed searches for observational studies reporting bisphosphonates use and AF with the electronic databases described in the Method section and identified 71 potential citations. After exclusion of RCTs (n = 4), unrelated observational studies (n = 28), repeated quotations (11), review articles (n = 8), author replies (n = 10), case reports (n = 2) and non-English articles (n = 5), 3 population-based observational studies with case-control design were eligible for analysis [19-21]. Figure 1 summarizes the results of the literature search.

### Summary of the RCTs

For the 4 eligible RCTs (the HORIZON Pivotal Fracture Trial, the HORIZON Recurrent Fracture Trial and the 2 FIT), 8,152 patients were randomly assigned to receive bisphosphonates while 8,132 patients received placebo. For the combined results of the 5 RCTs on risedronate and 4 on ibandronate, 16,848 were randomized to receive bisphosphonates while 6,972 were not. Table 1 summarizes the characteristics of the RCTs included in the meta-analysis.

**Table 1:** Characteristics and quality of randomized control trials comparing bisphosphonates users and controls with regard to atrial fibrillation

**Author, year**	*Studies included in reports (Ref)	**Publication**	**N**	**†AF/BP users versus** **AF/non-BP users**	**Age** **(mean)**	**Study duration** **(years)**	**Jadad** **score** **(1-5)**
Black, 2007	NA	Full text	7714	94/3862 versus 73/3852	73	3	4

Cummings, 2007	Black, 1996^a ^(27)	Letter to the	2027	81/3236 versus	70.8	3	4
	Cummings, 1998^a ^(28)	editor	4432	71/3223	67.7	4.2	3

Lyles, 2007	NA	Full text	2111	29/1054 versus 27/1057	74.5	5	4

Karam, 2007	Combination of trials ^b^	Letter to the editor	15066	Summary: 189/10018 versus 94/5048	73.5	NA	NA

Papapoulous, 2008	Chesnut, 2005 (30)		2929		68.7	3	3
	Recker, 2004 (31)	Abstract	2860	Summary:	67	3	4
	Reginster, 2006 (32)		1583	57/6830 versus 18/1924	66	2	4
	Eisman, 2008 (33)		1382		65.9	2	4

*Randomized controlled trials included in letters to the editor and abstracts

† Number of cases of AF in bisphosphonate users versus number of cases of AF in non-bisphosphonate users

^a ^Two Fracture Intervention Trials

^b ^Involved trials: Vertebral Efficacy with Risedronate Therapy Trial, the Bone Mineral Density Trial, the Risedronate

5 mg Daily Prevention Trial, the Glucocorticosteroid - Induced Osteoporosis Prevention and Treatment Trials and the

Hip Intervention Program Trial

Abbreviations: Ref, references; N, number of patients; AF, atrial fibrillation; BP, bisphosphonates; NA, not applicable

Data of AF reported in the two FIT [30,31] were combined and published in the form of a report [17]. Since patients who participated in the FIT were captured in the same region and study centres, were postmenopausal female and observed for a similar mean observation period, the 2 studies of FIT would have contributed comparable variance if they were separately entered into the meta-analysis. Therefore we decided to assign this report for analysis as 2 separate RCTs. On the contrary, we did not assign the 2 reports of RCTs involving risedronate [18] and ibandronate [32] for analysis with the two HORZION trials [11,16] and the two FIT studies [30,31] because (i) the risedronate and ibandronate studies were of different designs and some of them compared the efficacy and safety of widely different dosages of risedronate and ibandronate [33,34], and (ii) some of these studies did not include a placebo group [35,36]. Considerable heterogeneity between these risedronate and ibandronate trials is therefore expected and the variance of each of these trials is unknown because only combined, rather than data of individual study, was reported. We therefore decided that rather than combining all the results of the RCTs, it would be more appropriate to perform a sensitivity analysis to test the robustness of the result of the 4 RCTs (the HORIZON Pivotal Fracture Trial, the HORIZON Recurrent Fracture Trial and the other is a report of the 2 FIT) by comparing with the combined RRs of these 4 RCTs and the 2 reports respectively on risedronate (consisting of 5 RCTs) and ibandronate (consisting of 4 RCTs).

### Summary of the observational studies

In the 3 retrospective population-based observational case-control studies [19-21], 18,251 patients received bisphosphonates while 107,792 were not exposed to bisphosphonates. Two studies retrospectively identified bisphosphonate users in patients with AF in healthcare delivery system setting and national registries respectively [19,20] while the other identified AF in patients and controls with fractures who received bisphosphonates [21]. Although the initial patients of target were different between these observational studies, their study design and aim were similar. Table 2 summarizes the characteristics of the eligible population-based case-control studies.

**Table 2:** Characteristics of population based case-control studies comparing bisphosphonates users and controls with regard to atrial fibrillation

**Author, year**	**Publication**	**N**	**†AF/BP users versus** **AF/non-BP users**	**Age (mean)**	**Study duration (years)**
Heckbert, 2008	Full text	1685	47/87 versus 672/1598	72.7	3*
Sorensen, 2008	Full text	81505	724/3862 versus 12862/77643	76.1	6**
Abrahamsen, 2009	Full text	43033	797/14302 versus 1280/28731	74.3	10***

† Number of cases of AF in bisphosphonate users versus number of cases of AF in non-bisphosphonate users

* Atrial fibrillation occurred between 1^st ^October 2001 and 31^st ^December 2004

** Atrial fibrillation occurred between 1999 and 2005

***Atrial fibrillation occurred between 1^st ^January 1996 and 31^st ^December 2005

Abbreviations: N, number of subjects in the study; AF, atrial fibrillation; BP, bisphosphonates

### Calculation of the rates of AF in bisphosphonates users and non-bisphosphonate users

Unlike the RCTs which clearly stated the number of patients in the treatment groups and placebo groups who developed AF, the occurrence of AF in bisphosphonate users and non-users in the observational case-control studies were not explicitly stated. Based on the data of each observation study, we calculated the occurrence of AF in bisphosphonates users and non-users as follows: (i) For Herbeck's study [19], the number of alendronate users was 87 (47+40). Amongst these 87 patients who were exposed to the bisphosphonate, 40 of them developed AF. For those who were not exposed to alendronate (672+926 = 1,598), 672 developed AF. (ii) For Sørensen's study [20], the number of patients who were ever exposed to bisphosphonates was 3,862 (435+289+1,958+1,180). Amongst these 3,862 patients who were exposed to bisphosphonates, 724 (435+289) of them developed AF. For those who were not exposed to bisphosphonate (12862+64781 = 77,643), 12,862 of them developed AF. (3) For Abrahamsen's study [21], the number of patients with AF is equal to the number of patients × incidence of AF (1000 patient years) × mean follow-up time. Therefore the occurrence of AF in bisphosphonate users = (20.6/1000) × 2.7 × 14,302 = 797, while that of non-bisphosphonate users = (16.5/1000) × 2.7 × 28731 = 1,280.

### Combined OR of AF from 4 RCTs

**Figure 2:**
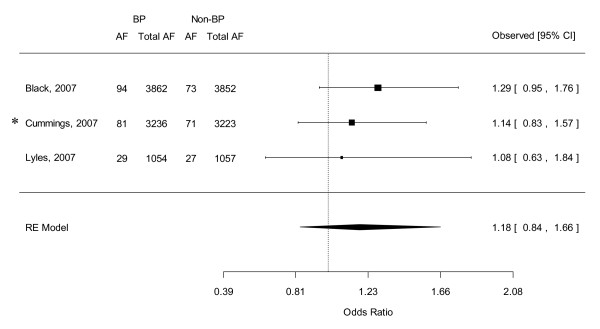
Forest plot of the odds ratio of atrial fibrillation: Bisphosphonate users versus controls by pooling *4 RCTs. Abbreviations: RCT, randomized control trial; BP, bisphosphonate; AF, atrial fibrillation; CI, credible interval; RE, random effects. *Two RCTs in Cummings et al's study. See text for details.

Data from 4 RCTs (the HORIZON Pivotal Fracture Trial, the HORIZON Recurrent Fracture Trial and the 2 FIT of alendronate) were pooled and the combined estimate revealed a non-significantly higher risk of AF amongst patients in the bisphosphonate group compared with those in the placebo group (OR 1.184, 95% CI 0.837-1.656) (See Figure 2). As predicted in our assumption discussed above, I^2 ^statistics showed that these studies were very homogeneous (*Q*(*df *= 2) = 0.466, *p *= 0.792, I^2 ^= 0). Egger's regression test revealed no evidence of significant publication bias in these RCTs (Intercept = -1.196, standard error (SE) = 1.368, p = 0.543, 2 tailed).

### Combined OR of serious AF from 4 RCTs

**Figure 3:**
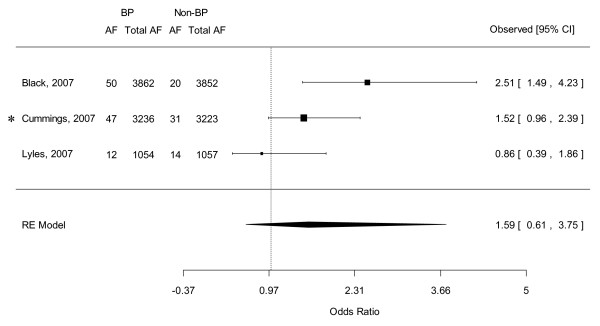
Forest plot of odds ratio of serious atrial fibrillation: Bisphosphonate users versus controls by pooling *4 RCTs. Abbreviations: RCT, randomized control trial; BP, bisphosphonate; AF, atrial fibrillation; CI, credible interval; RE, random effects. * Two RCTs in Cummings et al's study. See text for details.

Cases of serious AF were reported in the RCTs when patients with AF resulted in hospitalization, disability or judged to be life threatening [11,16]. When we combined the RRs of serious AF cases reported in the 4 RCTs (the HORIZON Pivotal Fracture Trial, the HORIZON Recurrent Fracture Trial and the 2 FIT of alendronate), patients who had bisphosphonates again demonstrated a non-significantly higher risk of serious AF compared with those received placebo (OR 1.590, 95% CI 0.613-3.751) (see Figure 3). Heterogeneity was relatively substantial as assessed by the I^2 ^statistics (*Q*(*df *= 2) = 5.368, *p *= 0.068, I^2 ^= 62.7). Publication bias was not evident as estimated by Egger's regression test (Intercept = -3.896, SE = 5.207, p = 0.591, 2 tailed).

### Combined OR of AF from observational studies

**Figure 4:**
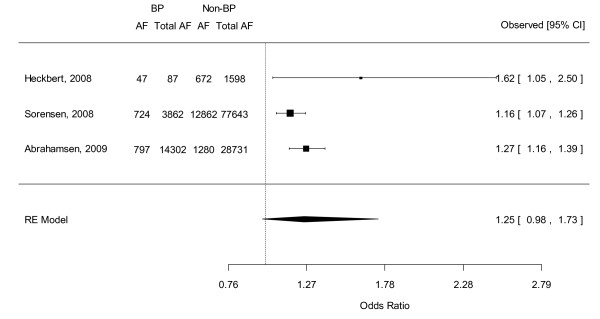
**Forest plot of the odds ratio of atrial fibrillation: Bisphosphonate users versus non-bisphosphonate users by pooling 3 observational studies**. Abbreviations: BP, bisphosphonate; AF, atrial fibrillation; CI, credible interval; RE, random effects.

Combination of the results of the three large population-based case-control studies revealed that bisphosphonate users had a non-significantly higher risk of development of AF compared to non-bisphosphonate users (OR 1.251, 95% CI 0.980-1.732). Heterogeneity between these 3 studies were mild (*Q*(*df *= 2) = 3.572, *p *= 0.168, I^2 ^= 44.0) (see Figure 4). Egger's regression test revealed no evidence of significant publication bias in these observational studies (Intercept = 1.782, SE = 1.574, p = 0.461, 2 tailed).

### Combined OR for observational studies and RCTs

According to Sutton et al and Ades at el [24,25], the pooled OR and its sampling variance from the observational studies could be used as the prior in the analysis of RCTs. Ten times the sampling variance of the pooled OR obtained from the observational studies was used as the variance of the pooled OR in the prior. This provides a conservative estimate on the pooled OR and its CI. The pooled effect size and its 95% CI was OR 1.194, 95% CI 0.923-1.540 by combining RCTs with observational studies. Besides reporting the statistical significance of the effect size, clinicians are particularly interested in the practical significance of the findings which indicates whether the effect size is large enough to be of value in a practical point of view. The value of practical significance depends on the specific context. For example, if we define OR>1.2 as the practical significance, by checking the posterior probability, the estimated probability for OR>1.2 was 0.484. This indicates that the probability of observing a study with OR>1.2 is about 0.48.

### Sensitivity analysis

The sensitivity analysis comprised two parts. First, we confirmed the robustness of the combined OR of the 4 RCTs (the HORIZON Pivotal Fracture Trial, the HORIZON Recurrent Fracture Trial and the 2 FIT of alendronate) by comparing it with the OR pooling from these 4 RCTs with the 2 reports of RCTs (5 on risedronate and 4 on ibandronate). Second, since the results of Bayesian meta-analysis are sensitive to the priors, we used several priors to check the sensitivity of the results. To address the sensitivity of the priors on the results, two priors based on the variance of the pooled OR of the observational studies were used. They were the sampling variance of the pooled OR and 10 times of it. The first prior can be considered as a liberal prior because it puts more weight on the observational studies. The second prior is more conservative as it puts less weight on the observational studies.

**Figure 5:**
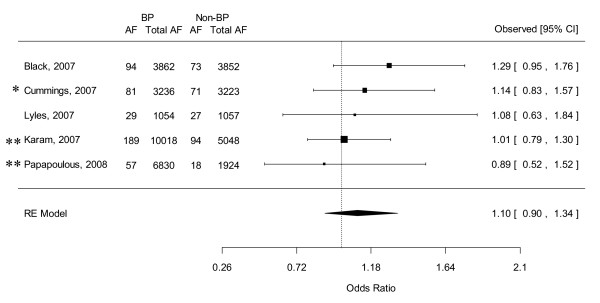
Forest plot of the odds ratio of atrial fibrillation: Bisphosphonate users versus controls by pooling *4 RCTs and **2 reports of RCTs. Abbreviations: RCT, randomized control trial; BP, bisphosphonate; AF, atrial fibrillation; CI, credible interval; RE, random effects. * Two RCTs in Cummings et al's study. **Karam 2007 and Papapoulous 2008 are 2 reports of RCTs. See text for details

The result remained robust when the 4 RCTs (the HORIZON Pivotal Fracture Trial, the HORIZON Recurrent Fracture Trial and the 2 FIT of alendronate) and the 2 reports of RCTs (5 on risedronate and 4 on ibandronate) were combined (OR 1.096, 95% CI 0.896-1.342). No notable publication bias was noted (Intercept = -0.477, SE = 1.301, p = 0.738, 2 tailed). (See Figure 5 and Table 3).

**Table 3:** Result of sensitivity analyses by comparing the combined ORs of *all RCTs and of **4 RCTs

	**Analysis using all RCTs**			**Analysis using 4 RCTs**		
**Outcome**	**OR (95% CI)**	***df***	**I**^2^	**OR (95% CI)**	***df***	**I**^2^
Atrial fibrillation	1.096 (0.896 - 1.342)	4	0.000	1.184 (0.837 - 1.656)	2	0.000

The pooled ORs and their 95% CIs for the liberal and the conservative priors were close, which are OR 1.222, 95% CI 1.119-1.335 and OR 1.194, 95% CI 0.923-1.540, respectively. The posterior probabilities of different values of the practical significance (from OR>1.1 to OR>1.5) were shown in Table 4. Clinicians may refer to the probability for the desired level of practical significance.

**Table 4:** Posterior probability of effect sizes larger than specific odds ratio

**Odds ratio**	*Liberal prior: mu~dnorm(0.2044, 430)	*Conservative prior: **mu ~dnorm(0.2044, 10)**
>1.1	.990	.753

>1.2	.655	.484

>1.3	.084	.241

>1.4	.001	.098

>1.5	.000	.037

*Prior distribution was obtained from the posterior distribution of the Bayesian meta-analysis on the observational studies. The inverse of the estimated variance was used as the prior in the liberal prior condition while the inverse of ten times of the estimated variance was used as the prior in the conservative condition.

## Discussion

Meta-analysis of RCTs revealed only a trend of higher AF risk in bisphosphonate users compared with patients on placebo. The reasons for this non-significant result are many. First, these RCTs might lack sufficient power to detect such a rare occurrence of AF. Second, the previously reported association between bisphosphonate use and AF might simply be due to chance, as so far only one RCT could demonstrate a significant risk of serious AF associated with bisphosphonate use [16] while other studies either showed a non-significant trend [17] or even contradictory result [11]. Thirdly, although meta-analysis is a powerful statistical method to combine study data and generate an effect size with a greater power, it might not be the best way to draw conclusion especially when meta-analysis is based on just a few studies.

The discrepancy in the effect size between overall and serious AF warrants further discussion. At a closer look at individual RCT, the odds ratios varied widely between studies with evidence of opposing directions of the risk [11,16,17]. The reason of such inconsistency is unclear although discrepancies in the definition, detection and reporting of serious AF may play a role. A much higher heterogeneity (I^2 ^= 62.7) when effect size of serious AF was pooled compared with that of overall AF (I^2 ^= 0) might partially explain the possibility we stated.

Similarly, pooling the 3 population-based studies demonstrated that bisphosphonate users had a non-significantly higher risk than non-bisphosphonate users for developing of AF. Results obtained by pooling observational studies need to be interpreted with caution in that observation studies are limited by potential confounders which could not be eliminated when the data were combined in meta-analysis. Notably, the risk of AF amongst bisphosphonate users was higher in those with diabetes mellitus and statin use [19] and likewise, bisphosphonate users were noted to have a higher rate of antithrombotic and antihypertensive use in another study [21]. As these factors share a common risk for atrial fibrillation and osteoporosis which warrants bisphosphonate use, this further signifies the presence of potential residual confounding which may bias the results, even though extensive matching between cases and controls were performed in these observational studies.

Although statistical significance is important in addressing whether an OR is statistically different from unity, it would be more clinically relevant if clinicians are informed of the probability of certain risks of development of AF in bisphosphonate users. Bayesian meta-analysis was therefore the method of choice in this study because (i) it provides an appropriate statistical model to combine studies of RCTs and observational studies [24,25] and (ii) it offers the posterior probability which is practically relevant for clinicians. The major limitation of the Bayesian meta-analysis is that the results may be sensitive to the priors used. We thus attempted to test the sensitivity of the results by using different priors and our results showed that the pooled effect size is not sensitive to the priors used. The point estimate is not very sensitive to the priors used while the CI and the posterior probabilities of the practical significance are slightly sensitive to the priors used. This is expected as the number of studies in this analysis is not large.

While bisphosphonates have been expected to cause gastrointestinal side effects and electrolyte disturbance such as hypocalcaemia, AF is a recently acknowledged and unanticipated potential adverse event of bisphosphonates. Despite being increasingly reported in the literature, the biological mechanism for AF related to bisphosphonate use is largely enigmatic. Two putative mechanisms have been proposed. First, hypocalcaemia secondary to bisphosphonates intake or infusions might be a trigger of AF [37]. Since the majority of AF occurred more than 1 month after bisphosphonate administration by which time the serum calcium level was normal and the bisphosphonate level was largely undetectable [16], AF secondary to hypocalcaemia appears unlikely. Even though a transient drop of serum calcium level after administration of bisphosphonates may happen, whether such a drop of serum calcium can trigger AF is again, elusive.

Alternatively, bisphosphonates, especially when administrated intravenously, can induce release of pro-inflammatory cytokines such as tumour necrosis factor-alpha (TNF-α), interleukins-1 and 6 from inflammatory cells. These inflammatory cytokines may cause remodelling of the atrium, tissue organisation, fibrosis and subsequent development of AF [38,39]. Because the majority of AF occurred at a considerable period of time after administration of bisphosphonates, the latter mechanism seems more probable. Without doubt, laboratory studies and human studies involving more patients are required to establish the possible pathological relationship between bisphosphonates and AF.

Although meta-analysis is a strong tool and we have taken the necessary precautions to detect publication bias and eliminate the effect of heterogeneity by using the random effects model for all the analyses, there are still limitations of the current study and some of them are intrinsic to meta-analysis. First, the occurrence of AF in patients who received bisphosphonates is low (incidence around 2%) and the duration of RCTs is short. Compounded with the insufficiency of patients participating in the trials, meta-analysis of RCTs might not be the best method to detect the possible association between bisphosphonate use and such a rare event as AF. Second, the current meta-analysis was based on a few studies only and it is subjected to random error. Third, while publication bias was not significant statistically in these studies, such a bias can never be completely eliminated even though we have included publications in the forms of abstract and letters to the editor. Fourth, current evidence seems to advocate the possible role of bisphosphonate potency on the occurrence of AF [16,18,19,21]. In the present meta-analysis; however, we were not able to separately analyse the risk of AF for individual bisphosphonate because the dearth of data disallows meaningful analysis. For the same reason, the number of studies is insufficient to perform meaningful meta-regression analyses, a statistical procedure to detect covariate(s) which may explain the heterogeneity between studies. Fifth, AF was not determined prior to taking bisphosphonates in the majority of the patients, therefore whether the occurrence of AF was truly the result of bisphosphonate intake is unknown. Finally, in order to maintain homogeneity of the studies for the meta-analyses and minimize the confounding effect of the underlying conditions on the development of AF, we only included studies which evaluated the use of bisphosphonates in patients with bone loss and fractures. As a result, the cardiovascular adverse effects of bisphosphonate use in other conditions, such as malignancy, hypercalcaemia and metabolic bone diseases, were not assessed because these conditions may also be culprits of arrhythmias. Nevertheless, the precautions we implemented should render the current meta-analysis the latest available evidence to date which serves to alert clinicians to seriously consider the risk of AF and be informed the probability of certain risks of developing AF in their patients who are taking bisphosphonates.

While not being a primary objective of this study, the results underscore the potential limitation of meta-analysis of RCTs in detecting the association between treatment and rare treatment-related events. At this juncture; concurring with the FDA safety review report [40], physicians should not refrain from prescribing bisphosphonates to patients who are truly indicated for the medication. As AF can be potentially serious, physicians must be alerted should their patients who are taking bisphosphonate develop new cardiovascular or respiratory symptoms which may be secondary to AF.

## Conclusion

Bisphosphonate use was not associated with a significantly higher risk of AF when RCTs and observational were collectively analyzed. Nevertheless, based on Bayesian meta-analysis, the posterior probabilities of development of AF was found to be 0.484 if the risk of AF, based on the effect size of the observational studies, was estimated to be more than 20%. The results of the current meta-analysis offer clinicians the practical probability of development of AF in patients who need bisphosphonates for the treatment of bone loss and corticosteroid induced osteoporosis.

## List of abbreviations

ACR: American College of Rheumatology; AF: atrial fibrillation; ARHP: Association of Rheumatology Health Professionals; BP: bisphosphonate; CI: credible interval; *df*: degree of freedom; FIT: Fracture Intervention Trials; MOOSE: meta-analysis of observational studies in epidemiology; OR: odds ratio; QUOROM: quality of reporting of meta-analysis; RCTs: randomized controlled trials; SE: standard error; TNF-α: tumour necrosis factor-alpha

## Competing interests

The authors declare that they have no competing interests.

## Authors' contributions

AM generated the idea of this study and participated in its design, coordination, literature search, statistical analyses and manuscript preparation. MWLC performed statistical analyses and provided expert biostatistical inputs. AAC took part in the literature search and generated all the Tables and Figures of this manuscript. RCH participated in manuscript preparation. CSL is the senior author who participated in manuscript preparation and supervision of the overall process of the study. All authors have read and approved the final manuscript before submission for peer review.

## Authors' Information

Dr Anselm **Mak **[MMedSc, FRCP(Edin)] is an Assistant Professor of Medicine and Consultant in Rheumatology. His research interest includes chronic inflammation and organ damage in SLE, as well as meta-analysis. Dr Mike W.L-. **Cheung **(PhD) is an Assistant Professor and he is an expert in biostatistics and meta-analysis. Dr Roger Chun-man **Ho **(DPM, MRCPsych) is an Assistant Professor of Psychiatry and Associate Consultant in Psychiatry. His research interest is depressive disorders and meta-analysis. Miss Alicia Ai-Cia Cheak (BSc) is the chief study coordinator under Dr Anselm Mak's research team. Professor Chak Sing **Lau **(MD, FRCP) is a Professor of Rheumatology. His research interest is pathogenesis and vascular biology of SLE.

## Pre-publication history

The pre-publication history for this paper can be accessed here:



## References

[B1] Olszynski WP, Davison KS (2008). Alendronate for the treatment of osteoporosis in men. Expert Opin Pharmacother.

[B2] Wells GA, Cranney A, Peterson J, Boucher M, Shea B, Robinson V, Coyle D, Tugwell P (2008). Alendronate for the primary and secondary prevention of osteoporotic fractures in postmenopausal women. Cochrane Database Syst Rev.

[B3] Wells G, Cranney A, Peterson J, Boucher M, Shea B, Robinson V, Coyle D, Tugwell P (2008). Risedronate for the primary and secondary prevention of osteoporotic fractures in postmenopausal women. Cochrane Database Syst Rev.

[B4] Canalis E, Mazziotti G, Giustina A, Bilezikian JP (2007). Glucocorticoid-induced osteoporosis: Pathophysiology and therapy. Osteoporos Int.

[B5] Devogelaer JP, Goemaere S, Boonen S, Body JJ, Kaufman JM, Reginster JY, Rozenberg S, Boutsen Y (2006). Evidence-based guidelines for the prevention and treatment of glucocorticoid-induced osteoporosis: A consensus document of the Belgian Bone Club. Osteoporos Int.

[B6] Orwoll E, Ettinger M, Weiss S, Miller P, Kendler D, Graham J, Adami S, Weber K, Lorenc R, Pietschmann P, Vandormael K, Lombardi A (2000). Alendronate for the treatment of osteoporosis in men. N Engl J Med.

[B7] Adachi JD, Saag KG, Delmas PD, Liberman UA, Emkey RD, Seeman E, Lane NE, Kaufman JM, Poubelle PEE, Hawkins F, Correa-Rotter R, Menkes CJ, Rodriguez-Portales JA, Schnitzer TJ, Block JA, Wing J, McLlwain HH, Westhovens R, Brown J, Melo-Gomes JA, Gruber BL, Yanover MJ, Leite MOR, Siminoski KG, Nevitt MC, Sharp JT, Malice MP, Dumortier T, Czachur M, Carofano W (2001). Two-year effects of alendronate on bone mineral density and vertebral fracture in patients receiving glucocorticoids: A randomized, double-blind, placebo-controlled extension trial. Arthritis Rheum.

[B8] Ringe JD, Faber H, Farahmand P, Dorst A (2006). Efficacy of risedronate in men with primary and secondary osteoporosis: Results of a 1-year study. Rheumatol Int.

[B9] Steinbuch M, D'Agostino RB, Mandel JS, Gabrielson E, McClung MR, Stemhagen A, Hofman A (2002). Assessment of mortality in patients enrolled in a risedronate clinical trial program: A retrospective cohort study. Regul Toxicol Pharmacol.

[B10] Mok CC, Tong KH, To CH, Siu YP, Ma KM (2008). Risedronate for prevention of bone mineral density loss in patients receiving high-dose glucocorticoids: A randomized double-blind placebo-controlled trial. Osteoporos Int.

[B11] Lyles KW, Colon-Emeric CS, Magaziner JS, Adachi JD, Pieper CF, Mautalen C, Hyldstrup L, Recknor C, Nordsletten L, Moore KA, Lavecchia C, Zhang J, Mesenbrink P, Hodgson PK, Abrams K, Orloff JJ, Horowitz Z, Eriksen EF, Boonen S (2007). Zoledronic acid and clinical fractures and mortality after hip fracture. N Engl J Med.

[B12] Rizzoli R, Burlet N, Cahall D, Delmas PD, Eriksen EF, Felsenberg D, Grbic J, Jontell M, Landesberg R, Laslop A, Wollenhaupt M, Papapoulos S, Sezer O, Sprafka M, Reginster JY (2008). Osteonecrosis of the jaw and bisphosphonate treatment for osteoporosis. Bone.

[B13] Yarom N, Yahalom R, Shoshani Y, Hamed W, Regev E, Elad S (2007). Osteonecrosis of the jaw induced by orally administered bisphosphonates: Incidence, clinical features, predisposing factors and treatment outcome. Osteoporos Int.

[B14] Yanik B, Turkay C, Atalar H (2007). Hepatotoxicity induced by alendronate therapy. Osteoporos Int.

[B15] Coleman CI, Perkerson KA, Lewis A (2004). Alendronate-induced auditory hallucinations and visual disturbances. Pharmacotherapy.

[B16] Black DM, Delmas PD, Eastell R, Reid IR, Boonen S, Cauley JA, Cosman F, Lakatos P, Ping CL, Man Z, Mautalen C, Mesenbrink P, Hu H, Caminis J, Tong K, Rosario-Jansen T, Krasnow J, Hue TF, Sellmeyer D, Eriksen EF, Cummings SR (2007). Once-yearly zoledronic acid for treatment of postmenopausal osteoporosis. N Engl J Med.

[B17] Cummings SR, Schwartz AV, Black DM (2007). Alendronate and atrial fibrillation [25]. N Engl J Med.

[B18] Karam R, Camm J, McClung M (2007). Yearly zoledronic acid in postmenopausal osteoporosis [3]. N Engl J Med.

[B19] Heckbert SR, Li G, Cummings SR, Smith NL, Psaty BM (2008). Use of alendronate and risk of incident atrial fibrillation in women. Arch Intern Med.

[B20] Sorensen HT, Christensen S, Mehnert F, Pedersen L, Chapurlat RD, Cummings SR, Baron JA (2008). Use of bisphosphonates among women and risk of atrial fibrillation and flutter: Population based case-control study. BMJ.

[B21] Abrahamsen B, Eiken P, Brixen K (2009). Atrial fibrillation in fracture patients treated with oral bisphosphonates. J Intern Med.

[B22] Egger M, Smith GD, Phillips AN (1997). Meta-analysis: Principles and procedures. BMJ.

[B23] Jadad AR, Moore RA, Carroll D, Jenkinson C, Reynolds DJM, Gavaghan DJ, McQuay HJ (1996). Assessing the quality of reports of randomized clinical trials: Is blinding necessary?. Control Clin Trials.

[B24] Sutton AJ, Abrams KR (2001). Bayesian methods in meta-analysis and evidence synthesis. Stat Methods Med Res.

[B25] Ades AE, Sutton AJ (2006). Multiparameter evidence synthesis in epidemiology and medical decision-making. J R Statist Soc A.

[B26] Spiegelhalter DJ, Thomas A, Best NG, Lunn D (2001). WinBUGS user manual: version 1.4.

[B27] R Development Core Team (2009). R: A language and environment for statistical computing. http://www.r-project.org.

[B28] Moher D, Cook DJ, Eastwood S, Olkin I, Rennie D, Stroup DF (1999). Improving the quality of reports of meta-analyses of randomised controlled trials: The QUOROM statement. Lancet.

[B29] Stroup DF, Berlin JA, Morton SC, Olkin I, Williamson GD, Rennie D, Moher D, Becker BJ, Sipe TA, Thacker SB (2000). Meta-analysis of observational studies in epidemiology: A proposal for reporting. JAMA.

[B30] Black DM, Cummings SR, Karpf DB, Cauley JA, Thompson DE, Nevitt MC, Bauer DC, Genant HK, Haskell WL, Marcus R, Ott SM, Torner JC, Quandt SA, Reiss TF, Ensrud KE (1996). Randomised trial of effect of alendronate on risk of fracture in women with existing vertebral fractures. Lancet.

[B31] Cummings SR, Black DM, Thompson DE, Applegate WB, Barrett-Connor E, Musliner TA, Palermo L, Prineas R, Rubin SM, Scott JC, Vogt T, Wallace R, John Yates A, Lacroix AZ (1998). Effect of alendronate on risk of fracture in women with low bone density but without vertebral fractures. Results from the fracture intervention trial. JAMA.

[B32] Papapoulous S, Thompson L, Hartl F (2008). Ibandronate is not associated with an increased risk of atrial fibrillation; an assessment of annual cumulative exposure. Ann Rheum Dis.

[B33] Chesnut CH, Ettinger MP, Miller PD, Baylink DJ, Emkey R, Harris ST, Wasnich RD, Watts NB, Schimmer RC, Recker RR (2005). Ibandronate produces significant, similar antifracture efficacy in North American and European women: New clinical findings from BONE. Curr Med Res Opin.

[B34] Recker R, Stakkestad JA, Chesnut Iii CH, Christiansen C, Skag A, Hoiseth A, Ettinger M, Mahoney P, Schimmer RC, Delmas PD (2004). Insufficiently dosed intravenous ibandronate injections are associated with suboptimal antifracture efficacy in postmenopausal osteoporosis. Bone.

[B35] Reginster JY, Adami S, Lakatos P, Greenwald M, Stepan JJ, Silverman SL, Christiansen C, Rowell L, Mairon N, Bonvoisin B, Drezner MK, Emkey R, Felsenberg D, Cooper C, Delmas PD, Miller PD (2006). Efficacy and tolerability of once-monthly oral ibandronate in postmenopausal osteoporosis: 2 Year results from the MOBILE study. Ann Rheum Dis.

[B36] Eisman JA, Civitelli R, Adami S, Czerwinski E, Recknor C, Prince R, Reginster JY, Zaidi M, Felsenberg D, Hughes C, Mairon N, Masanauskaite D, Reid DM, Delmas PD, Recker RR (2008). Efficacy and tolerability of intravenous ibandronate injections in postmenopausal osteoporosis: 2-Year results from the DIVA study. J Rheumatol.

[B37] Van Wagoner DR, Nerbonne JM (2000). Molecular basis of electrical remodeling in atrial fibrillation. J Mol Cell Cardiol.

[B38] Hewitt RE, Lissina A, Green AE, Slay ES, Price DA, Sewell AK (2005). The bisphosphonate acute phase response: Rapid and copious production of proinflammatory cytokines by peripheral blood γδ T cells in response to aminobisphosphonates is inhibited by statins. Clin Exp Immunol.

[B39] Aviles RJ, Martin DO, Apperson-Hansen C, Houghtaling PL, Rautaharju P, Kronmal RA, Tracy RP, Van Wagoner DR, Psaty BM, Lauer MS, Chung MK (2003). Inflammation as a Risk Factor for Atrial Fibrillation. Circulation.

[B40] FDA Early Communication of an Ongoing Safety Review. http://www.fda.gov/Cder/Drug.

